# The Health and Welfare of Pigs from the Perspective of Post Mortem Findings in Slaughterhouses

**DOI:** 10.3390/ani10050825

**Published:** 2020-05-09

**Authors:** Vladimir Vecerek, Eva Voslarova, Zbynek Semerad, Annamaria Passantino

**Affiliations:** 1 Department of Animal Protection and Welfare and Veterinary Public Health, Faculty of Veterinary Hygiene and Ecology, University of Veterinary and Pharmaceutical Sciences Brno, 612 42 Brno, Czech Republic; vecerekv@vfu.cz; 2 Central Veterinary Administration of the State Veterinary Administration, 120 00 Prague, Czech Republic; z.semerad@svscr.cz; 3 Department of Veterinary Sciences, University of Messina, Polo Universitario Annunziata, 981 68 Messina, Italy; passanna@unime.it

**Keywords:** finisher pig, cull sow, piglet, slaughter, post mortem examination, health

## Abstract

**Simple Summary:**

The health and welfare of pigs was evaluated on the basis of the data on patho-anatomic findings obtained during the veterinary examination of pigs slaughtered in slaughterhouses in the Czech Republic in the period from 2010 to 2017. The highest numbers of lesions in organs were found in lungs (finisher pigs 41%, sows 24% and piglets 52%), kidneys (finisher pigs 14%, sows 32% and piglets 15%) and liver (finisher pigs 12%, sows 18% and piglets 19%). The character of most findings was chronic, however, acute findings were also detected. Parasitic lesions were found mainly in finisher pigs (finisher pigs 4%, sows 1% and piglets 1%). Incidence of traumatic lesions (finisher pigs 0.08%, sows 0.14% and piglets 0.15%) was far below the frequency of other findings. Overall, post mortem findings in the slaughterhouses varied among pig categories (*p* < 0.001). In order to decrease the number of lesions detected post mortem, it is essential to improve health and welfare of pigs on farms and in transit.

**Abstract:**

The health and welfare of pigs was evaluated on the basis of the data on patho-anatomic findings obtained during the veterinary examination of pigs slaughtered in slaughterhouses in the Czech Republic in the period from 2010 to 2017. High numbers of lesions in organs found especially in lungs (finisher pigs 41%, sows 24% and piglets 52%), kidneys (finisher pigs 14%, sows 32% and piglets 15%) and liver (finisher pigs 12%, sows 18% and piglets 19 %) indicate impaired health and welfare of pigs transported for slaughter. The differences in the number of findings between finisher pigs, sows and piglets were statistically significant (*p* < 0.001). The character of most findings was chronic, which document health and welfare problems occurring on farms as a result of the current pig husbandry. However, acute findings were also detected and indicated processes occurring shortly before and during transport to the slaughterhouse. An important finding is the incidence of parasitic lesions in the liver in finisher pigs (finisher pigs 4%, sows 1% and piglets 1%) that documents persistent occurrence of parasitic invasions on pig farms. Findings of traumatic lesions on limbs in sows and piglets (finisher pigs 0.08%, sows 0.14% and piglets 0.15%) are far below the frequency of the findings in organs; however, their incidence should be further reduced by adjusting the technology of housing, transport and handling. In conclusion, the level of health and related welfare of pigs based on the assessment of post mortem findings in the slaughterhouses vary. Overall, the worst situation is in piglets, followed by sows and the best evaluated are finisher pigs. Post mortem inspection revealed significant numbers of patho-anatomic changes even in pigs considered fit to be transported to the slaughterhouse and slaughtered for human consumption. It is clear that there is still a considerable space for improving the level of health and welfare of the individual categories of pigs.

## 1. Introduction

From the perspective of veterinary care, an essential requirement for housing and treatment of livestock is that the care provided to animals ensures their good health and welfare. The findings detected during the post mortem examination of pigs in slaughterhouses indicate changes that occur in the animals due to a disease or poor welfare. The analysis of the slaughterhouse data can contribute to the knowledge of the level of health and welfare of pigs and identification of health and welfare problems.

Several studies presented a retrospective analysis of the results of veterinary meat inspection at slaughterhouses concerning the rates of patho-anatomic changes in pig carcasses. Eckhardt et al. [1] and Kofer et al. [2] analyzed the results of the post-mortem inspection of pigs slaughtered at Austrian slaughterhouses. Both studies reported pneumonia, pleurisy, pericarditis and liver lesions among the most frequent findings. In a similar study carried out in Italy, Ceccarelli et al. [3] found most lesions in the liver, heart and pluck. Among the diseases, parasitic infestation of liver (ascariasis and distomatosis) was the most frequently present, followed by pericarditis and polyserositis. In Poland, tuberculosis, septicemia and pyemia, neoplasms, leukemia, icterus and emaciation were most often detected during pre- and post-slaughter inspection of pigs [4,5]. In total, pathological lesions were diagnosed in 41.43% [4] and 49.09% [5] of pig carcasses.

The impact of different systems of pig husbandry on the incidence of patho-anatomic findings detected during a slaughterhouse inspection was investigated by Hansson et al. [6] in Sweden and Kongsted and Sorensen [7] in Denmark. In conventionally reared finisher pigs, pleuritis, ascaris in the liver, abscesses and wounds on the tail due to tailbiting were detected more frequently whereas in pigs reared according to organic standards, joint diseases such as arthritis and arthrosis were found more often [6]. In pigs raised under free-range conditions (both organic and conventional), a higher incidence of different types of lesions was found than in pigs kept indoors. Specifically, a difference in the incidence of white liver spots, tail lesions, skin lesions, arthritis, bone fractures, septicaemia and abscesses was found. In contrast, the occurrence of leg swellings, hernia and hoof abscesses was more common in pigs reared in conventional indoor systems compared to free range systems [7].

Assumedly, the different categories of pigs (finisher pigs, cull sows and boars, piglets) slaughtered in the slaughterhouse will also differ in the incidence of patho-anatomic findings. This has, to the best of the authors’ knowledge, not been presented in the literature before.

The aim of the study was to assess which organs and body parts of pigs are damaged during the present intensive housing and handling of pigs in relation to pig health and welfare, on the basis of data obtained in the framework of veterinary supervision and inspection during slaughtering pigs in slaughterhouses in the Czech Republic in the period from 2010 to 2017.

## 2. Materials and Methods 

The assessment of the level of damage to organs and body parts of pigs housed in intensive husbandry systems (finisher pigs, sows and piglets) was based on the incidence of patho-anatomic findings in pigs reared in the Czech Republic and slaughtered in slaughterhouses in the Czech Republic in the period from 2010 to 2017. Only animals considered fit to be transported to the slaughterhouse (according to the Council Regulation (EC) No 1/2005 [8]) and slaughtered for human consumption were included in the analysis. The dataset consisted of 20,550,072 finisher pigs, 484,710 sows and 94,279 piglets, i.e., all pigs slaughtered in 257 slaughterhouses in the Czech Republic in the monitored period. For the purposes of this study, a ‘finisher pig’ means a pig that has reached the market weight, ‘sow’ means a female pig after the first farrowing and ‘piglet’ means a rearing pig from 10 weeks to premature slaughter (before reaching market weight). On farms, apart from some exceptions (farrowing sows and sows up to four weeks after the service) pigs were housed in groups in conventional pig houses on litter bedding. The transport of pigs from farms to slaughterhouses was carried out by means of road transport using trucks specifically designed for the transportation of pigs.

The data on the numbers of findings were obtained in cooperation with the State Veterinary Administration, which performs the official supervision in the slaughterhouses during slaughtering of pigs. The data on the numbers of animals slaughtered were obtained from a veterinary database collecting data on the transport and slaughter of food animals.

The classification of findings was based on the methodology for post mortem inspection of pigs. Specialized official veterinarians—veterinary inspectors—at slaughterhouses recorded during the inspection of individual carcasses the number of changes found in organs, i.e., liver and pancreas, intestines and stomach, lungs, heart, spleen, genitals, kidney, brain and spinal cord, skin, and in body parts, i.e., head, trunk and limbs. A separate category “overall changes” included findings involving the whole organism of the animal, such as insufficient development (dwarfism), emaciation, ascites, occurrence of abscesses on many organs, occurrence of tumors on many organs and others. Unclassified changes (such as findings on pleura, peritoneum, diaphragm, lymph nodes, hernia, unspecified morphological formations, color changes, developmental changes on organs, etc.) were recorded as “other unclassified changes”.

The findings on organs and body parts were further divided into acute, chronic, parasitic and traumatic changes. As acute changes were considered changes that showed signs of short-term intense inflammatory processes, i.e., with regard to the specificity of acute processes in individual organs, especially congestion, swelling, enlargement of the organ, presence of catarrhal, fibrinous, purulent or hemorrhagic exudate, etc.

As chronic changes were considered changes that showed signs of long-term inflammatory processes with further changes in the tissue, i.e., with regard to the specificity of chronic processes in individual organs, especially changes in the original tissue structure of the parenchyma accompanied by permeation of the connective tissue, with possible reduction of the organ, changes in the surface structure of mucous membranes and serous membrane layers in the sense of their coarsening, scar tissue and adhesions, postinflammatory cavities, cysts, tumors, etc.

Parasitic changes were not included in acute or chronic changes but were recorded separately in order to determine the extent of parasitic invasions. Parasitic findings were changes typical of parasitic invasion of the liver (e.g., ascariasis—findings of scars in the liver after invasion of ascarids), lungs (e.g., lungworms—findings of worms in the lungs), heart and muscle (cysticercosis—findings of cysticerci in muscle), etc.

Traumatic changes were not included in acute or chronic changes but were recorded separately in order to determine the extent of the traumatic lesions as a part of animal welfare inspection. Findings of traumatic origin were considered to be changes (acute or chronic) represented by open wounds at various stages of healing, hematomas in the subcutaneous tissue and muscles, contusions, dislocations, fractures and other changes that could have been caused directly by husbandry technology, improper human handling or other animals.

The number of patho-anatomic findings in pigs was evaluated in total and for the category of finisher pigs, the category of sows and the category of piglets separately. The incidence of individual findings was expressed as a percentage of the total number of animals slaughtered in the category. The results obtained were compared among the individual categories of patho-anatomic findings and pig categories. For statistical comparison of frequencies, the Chi-square test was used to assess the significance in the 2 × 2 contingency tables with Yates correction for frequencies exceeding 5, or the correction by the Fisher’s exact test for frequencies below 5 using the statistical package Unistat 6.5 for Windows (Unistat Ltd., London, UK). 

## 3. Results

The results show that when comparing the incidence of patho-anatomic findings in the individual organs in pigs slaughtered in slaughterhouses irrespective of pig category, the highest frequency of patho-anatomic findings was found in the lungs (41.09%), followed by findings in the kidneys (14.56%), liver (11.76%), heart (7.87%), spleen (1.98%), intestines and stomach (1.74%) and genitals (0.33%). Incidence of patho-anatomic findings in skin and brain and spinal cord was less than 0.01%. When comparing the incidence of patho-anatomic findings in the individual body parts of pigs slaughtered in slaughterhouses, the highest frequency of patho-anatomic findings was found in the limbs (0.67%), followed by findings in the trunk (0.52%). Incidence of patho-anatomic findings in the head was less than 0.01%. The differences in the incidence of the individual patho-anatomic findings in the individual organs and body parts were statistically highly significant (*p* < 0.001).

The numbers of patho-anatomic findings in individual organs and body parts of slaughtered pigs are shown in Table 1. The incidence of patho-anatomic findings varied for the individual categories. Finisher pigs were found to have an incidence of patho-anatomic findings exceeding 10% in the lungs, kidneys and liver. In sows, the occurrence of patho-anatomic findings exceeding 10% was in the kidneys, liver, lungs and spleen and in piglets in the lungs, liver, heart and kidneys. When comparing the incidence of patho-anatomic findings in the individual body parts of pigs slaughtered in slaughterhouses, the highest frequency of patho-anatomic findings was found in the limbs in all pig categories. Significantly lower (*p* < 0.001) frequency of patho-anatomic findings was detected on the animals’ trunks and heads. The differences in the incidence of the individual patho-anatomic findings between the pig categories were statistically highly significant (*p* < 0.001).

A comparison of the numbers of the most frequent patho-anatomic findings on pig organs from the viewpoint of further breakdown into acute, chronic and parasitic changes in finisher pigs, sows and piglets is presented in Table 2, Table 3 and Table 4. The results show that in the lungs, mostly chronic changes were detected (Table 2). Additionally, for the kidneys, the changes with the highest frequency were chronic changes (Table 3). Furthermore, the highest frequency of chronic findings was reported in liver (Table 4). The incidence of parasitic findings was marginal in comparison with chronic and acute changes; although the frequency of parasitic findings in the liver in finisher pigs is noteworthy.

A comparison of the occurrence of individual groups of patho-anatomic findings in pigs slaughtered in slaughterhouses with the focus on limbs and trunk is shown in Table 5 and Table 6. A high frequency of chronic lesions (in comparison with acute and traumatic findings) was found on the limbs, the highest being in sows (Table 5). Additionally, among the findings on the trunk, the incidence of chronic findings was the highest (in comparison with acute and traumatic findings), in the piglet category showing the highest percentage (Table 6). Traumatic changes were more frequently found on the limbs than on the trunk and head in all pig categories.

When comparing the incidence of the individual groups of patho-anatomic findings in pigs slaughtered in slaughterhouses with the focus on the overall changes (Table 7), the most frequent incidence of insufficient development (dwarfism) was recorded in piglets. Furthermore, piglets were found to have the highest frequency of emaciation. Abscesses were most frequently found in sows.

## 4. Discussion

The incidence of patho-anatomic findings differed in finisher pigs, sows and piglets slaughtered in slaughterhouses and document the differences in health and welfare between monitored pig categories. In finisher pigs, a high incidence of patho-anatomic findings was found especially in the lungs, kidneys and liver. Similarly, Januskeviciene et al. [9] detected the highest number of lesions in the respiratory system and liver, followed by kidney pathologies. Respiratory system illnesses belong to the most problematic issues of indoor intensive systems in conventional swine production [6] and present also a major welfare problem due to its widespread nature and severe lung lesions found at slaughter. The husbandry system with large numbers of animals of the same category housed in confined spaces and fed with dry mixtures might explain that finisher pigs often suffer from lung diseases (as dust exposure increases susceptibility to viral and bacterial pulmonary diseases). Kofer et al. [2] reported that 43.7% of the inspected pigs showed various phases of pneumonia and 22.7% of the animals suffered from chronic pleuritis. According to Hansson et al. [6], a considerably higher extent of pleuritis was found in conventionally kept pigs in comparison to those from organically certified herds that are kept partially outdoors, in smaller groups and without mixing of stock. The most risky management factors that greatly contribute to the occurrence of pneumonia include crowding, ammonia levels, relative humidity, fluctuation in temperature and mixing of stock of different origin [6]. Furthermore, probably as a result of intensive feeding at the edge of metabolic capacity (feed with a high content of concentrated nutrients and intensive metabolism resulting in muscle mass growth), damage to liver and kidney may occur, being the metabolic organs. Ceccarelli et al. [3] who investigated the causes of the confiscation of carcasses and organs at a slaughterhouse in Central Italy from 2010 to 2016 found the liver to be the most confiscated organ in pigs. The second most confiscated organ was the heart. In our study, the patho-anatomic changes in a heart were more frequently found in piglets than in finisher pigs. Similarly, piglets as a result of the housing (a large number of young animals of the same category in confined spaces and feeding with dry mixtures with a dust impact on the respiratory system) may suffer from lung diseases. Intensive metabolism affects the liver and kidneys; the metabolic burden is also reflected in the frequency of the occurrence of patho-anatomic changes in the heart.

In sows, the patho-anatomic changes were found mainly in the kidneys, liver, lungs, spleen and genital tract. Sows, as a result of systems aimed at the maximum number of piglets produced, are burdened with intensive metabolism associated with pregnancy, delivery and lactation. They are exposed to metabolic loads with an impact on the parenchymatic organs of the liver, kidneys and spleen. Lung diseases have an impact on the lungs. In addition, they suffer from genital disorders. Kozak et al. [10] explain the significantly increased number of emergency slaughters in sows compared to other pigs due to the high burden on sows within breeding.

These differences in the level of organ infliction between finisher pigs, sows and piglets are also the result of the different reasons for sending these categories of pigs for slaughter. Finisher pigs are sent to slaughterhouses in a good physical and health condition corresponding to young animals with good nutrition. Sows are sent at the end of their breeding use corresponding to older animals with a lower health condition. According to de Jong et al. [11] and Zhao et al. [12], reproductive problems, reduced health and age are among the major reasons reported for culling. Correspondingly, cull sows (as compared with finisher pigs) were reported to make up the majority of swine arriving as fatigued, lame or in a very low body condition [13]. Piglets are sent to a slaughterhouse prior to their utilization, usually due to deficiencies in the physical and/or health condition (insufficient development, emaciation). The growing–fattening is the most expensive period of the pig’s life and removing piglets with a delayed growth is a strategy to reduce the production costs, as lighter piglets are not likely to catch up with their weightier conspecifics during subsequent phases of production as documented by Lopez-Verge et al. [14].

When comparing the incidence of patho-anatomic findings in the individual parts of pig bodies slaughtered in slaughterhouses, the highest frequency of patho-anatomic findings is shown on the limbs, especially in piglets and sows. These findings document the differences in the husbandry of the different categories of pigs. Finisher pigs housed in balanced groups of animals on deep-litter bedding are not burdened with a significant disruption of the musculoskeletal system, while sows are often affected by changes in the limbs and the body resulting from prolonged duration of being housed in systems burdening the musculoskeletal system (slats, hard floors causing pressure sores, etc.). The weight of sows also contributes to lesions at the joints and claws. There are a number of studies on the design of slatted concrete floors for smaller animals such as finisher pigs, however, it is still necessary to optimize the design of slatted floors in order to decrease the negative effect on sow feet and leg health [15]. One of the most frequently stated reasons for premature culling of breeding sows is lameness [16], which can also influence the overall health and welfare of the sow and thus predispose to a loss of body condition (lame sows may not be strong enough to fight for their food), shoulder lesions (caused by an increased lying time and inactivity) and urogenital infections (more sitting and lying lead to an increase in micro-organisms around the perineal region due to dirtiness) [17]. Lameness has been suggested to be an important animal-based indicator of well-being in pigs [18]. It reflects both problems in the environment, such as unsuitable flooring and social challenges, and a welfare problem in itself as it is a condition indicative of pain [19]. Acutely and severely lame sows are instantly excluded from the herd. However, also chronic, less severe lameness can influence the performance of sows, leading consequently to culling as well [17]. Likewise, some atypical piglets housed in systems adapted to the average-sized piglets may be affected by pressure sores, bruises and other disorders on the limbs and/or the body, particularly animals being in a worse condition. Beyer and Wechsler [20] assessed the influence of the type of floor on the claw condition of weaners and found that even a difference of 1 mm in the slat gap had an impact on forming and severity of claw lesions (wall bruising and coroner lesions). Moreover, injuries and skin lesions in piglets often result from the hierarchical fights among piglets as a result of mixing unfamiliar piglets at weaning [21]. Leg weakness often causes stress and pain to the pig, and also impairs the piglet´s ability to compete at the feeding through. Thus, leg weakness is related to several welfare factors [22].

From the results of the comparison of the most frequent patho-anatomic findings on pig organs (divided into acute, chronic and parasitic changes), the dominant origin of patho-anatomic findings in pig husbandry (chronic findings) is evident in all categories. However, a relatively high proportion of lesions in limbs and on the trunk of acute character in sows and piglets that occurred on farms shortly before or during transport was also found.

An important finding is the incidence of parasitic diseases in finisher pigs. Correspondingly, Ceccarelli et al. [3] found the presence of liver lesions attributable to *Ascaris suum* constantly throughout the period monitored in their study. Moving of ascarid larvae through the liver, lungs and other organs may provide a gateway for pathogenic micro-organisms and inflict lesions resulting in condemnation at slaughter [6]. In our study, the incidence of parasitic diseases in finisher pigs was higher than the incidence of parasitic findings in sows, which could be more likely expected due to longer housing time. However, there are probably less changes in group composition in the life of sows as they usually stay on the same farm during their productive lives whereas pigs are often mixed from different origins at the beginning of the fattening phase, so there is a constant risk of introduction of *Ascaris* eggs by different piglet breeders. Another explanation probably lies in the differences in the infestation of finisher pigs and sows farms by parasites (ascarids) and in the susceptibility to these parasitic invasions in young animals (finisher pigs) and older animals (sows). Older pigs tolerate parasitic settlement better and can suppress larval migration [6,23]. Furthermore, at the time of culling, some of the parasitic lesions are healed [6]. Additionally, immunity can occur after *Ascaris* infection [24].

The occurrence of findings of traumatic origin demonstrates the need for further improvement in the level of the treatment of pigs during housing and transport. The differences in the numbers of findings of traumatic origin on the limbs found during the slaughterhouse inspection of finisher pigs, sows and piglets correspond to the differences in mortality rates during their transport for slaughter. Significantly greater transport-related mortality was found in sows and piglets than in finisher pigs [25,26]. According to Averos et al. [27], the mortality rates significantly correlate with the proportion of injured pigs in transit. The occurrence of traumatic findings on the limbs in sows, piglets and finisher pigs document shortcomings in handling of animals during housing and transport. The varieties in barn design and in the handling of pigs being moved out of the barn and loaded on the truck influence the stress response, the occurrence of injuries, pig losses and also the quality of carcasses and meat [28,29]. Appropriate condition of pigs for transport is a major factor influencing the potential suffering of the animals during transport. Nevertheless, the present guidelines and regulations do not always ensure that only fit animals are transported [30]. Among the transported animals, some may also be wounded or injured during transport. Moreover, as Thodberg et al. [31] documented in cull sows transported to slaughter, several types of injuries including torn hoofs deteriorate significantly during transportation.

Worse condition and health of piglets and sows slaughtered in slaughterhouses is also evidenced by the prevalence levels of insufficient development (dwarfism), emaciation and abscesses in these categories compared to finisher pigs. According to Hansson et al. [6], abscesses might be secondary to wounds caused by a rough floor surface without sufficient bedding material. Sows spend a considerable amount of time in farrowing stalls and insemination units where bedding material is not provided. Slatted or solid floors without bedding are typically used also for housing weaner piglets. In piglets, tail biting and hence injuries might also lead to formation of abscesses. However, in the Czech Republic most piglets have their tails cut within the first week of age. The third route of entry as suggested by Hansson et al. [6] may be an injection site. Finisher pigs might have fewer injection sites where pathogens may be introduced and lead to the formation of abscesses than sows and piglets.

## 5. Conclusions

Despite only the pigs being considered fit to be transported to the slaughterhouse and slaughtered for human consumption (based on on-farm assessment of fitness for transport), post mortem examinations revealed frequent patho-anatomic findings suggesting health and welfare of the pigs being impaired. In terms of the frequency of the occurrence of patho-anatomic findings the worst-off are piglets, then sows and the best are finisher pigs. Overall, the results of post mortem inspection show the need for further improvement of pig housing and transport conditions in order to reduce the negative impact of intensive husbandry on pig organs and other body parts, thereby improving pig health and welfare.

## Figures and Tables

**Table 1:** Comparison of patho-anatomic findings by individual organs and body parts in finisher pigs, sows and piglets slaughtered in the slaughterhouses in the Czech Republic from 2010 to 2017.

Findings	Finisher Pigs (*n* = 20,550,072)		Sows (*n* = 484,710)		Piglets (*n* = 94,279)	
	Number	%	Number	%	Number	%
Liver and pancreas	2,377,364	11.57 ^c^	88,545	18.27 ^b^	19,258	20.43 ^a^
Intestines and stomach	341,313	1.66 ^c^	21,536	4.44 ^b^	5725	6.07 ^a^
Lungs	8,513,214	41.43 ^b^	118,854	24.52 ^c^	49,689	52.70 ^a^
Heart	1,618,884	7.88 ^b^	26,728	5.51 ^c^	17,210	18.25 ^a^
Spleen	359,022	1.75 ^c^	57,545	11.87 ^a^	2728	2.89 ^b^
Genitals	30,162	0.15 ^b^	40,394	8.33 ^a^	87	0.09 ^c^
Kidney	2,908,676	14.15 ^c^	153,663	31.70 ^a^	14,280	15.15 ^b^
Brain and spinal cord	17	0.83 × 10^−4 b^	8	16.50 × 10^−4 a^	0	0 ^a,b^
Skin	20,404	0.10 ^b^	312	0.06 ^c^	148	0.16 ^a^
Head	1490	0.73 × 10^−2 b^	183	3.78 × 10^−2 a^	13	1.38 × 10^−2 b^
Trunk	102,224	0.50 ^c^	5499	1.13 ^b^	1590	1.69 ^a^
Limbs	105,231	0.51 ^c^	29,296	6.04 ^b^	6149	6.52 ^a^
Overall changes	68,774	0.34 ^c^	14,027	2.89 ^b^	17,702	18.78 ^a^
Other unclassified changes	185,496	0.90 ^c^	38,580	7.96 ^b^	9121	9.67 ^a^

^a,b,c^ percentages within a row lacking a common superscript differ (*p* < 0.001).

**Table 2:** Comparison of the number of individual patho-anatomic findings in lungs in finisher pigs, sows and piglets slaughtered in the slaughterhouses.

Origin of Findings	Finisher Pigs (*n* = 20,550,072)		Sows (*n* = 484,710)		Piglets (*n* = 94,279)	
	Number	%	Number	%	Number	%
Acute	436,340	2.12 ^b,z^	27,456	5.66 ^b,y^	15,211	16.13 ^b,x^
Chronic	8,070,598	39.27 ^a,x^	91,235	18.82 ^a,z^	34,434	36.52 ^a,y^
Parasitic	6276	0.03 ^c,y^	163	0.03 ^c,x,y^	44	0.05 ^c,x^

^a–c^ percentages in columns lacking a common superscript differ (*p* < 0.001); ^x–z^ percentages in rows lacking a common superscript differ (*p* < 0.01).

**Table 3:** Comparison of the number of individual patho-anatomic findings in kidneys in finisher pigs, sows and piglets slaughtered in the slaughterhouses.

Origin of Findings	Finisher Pigs (*n* = 20,550,072)		Sows (*n* = 484,710)		Piglets (*n* = 94,279)	
	Number	%	Number	%	Number	%
Acute	55,965	0.27 ^b,z^	22,415	4.62 ^b,x^	3182	3.38 ^b,y^
Chronic	2,850,380	13.87 ^a,y^	131,137	27.05 ^a,x^	11,063	11.73 ^a,z^
Parasitic	2331	0.01 ^c,z^	111	0.02 ^c,y^	35	0.04 ^c,x^

^a–c^ percentages in columns lacking a common superscript differ (*p* < 0.001); ^x–z^ percentages in rows lacking a common superscript differ (*p* < 0.05).

**Table 4:** Comparison of the number of individual patho-anatomic findings in liver in finisher pigs, sows and piglets slaughtered in the slaughterhouses.

Origin of Findings	Finisher Pigs (*n* = 20,550,072)		Sows (*n* = 484,710)		Piglets (*n* = 94,279)	
	Number	%	Number	%	Number	%
Acute	41,065	0.20 ^c,z^	3924	0.81 ^b,y^	5272	5.59 ^b,x^
Chronic	1,554,729	7.57 ^a,z^	80,795	16.67 ^a,x^	12,619	13.38 ^a,y^
Parasitic	780,952	3.80 ^b,x^	3,774	0.78 ^b,z^	1364	1.45 ^c,y^

^a–c^ percentages in columns lacking a common superscript differ (*p* < 0.001); ^x–z^ percentages in rows lacking a common superscript differ (*p* < 0.001).

**Table 5:** Comparison of the number of individual patho-anatomic findings in limbs in finisher pigs, sows and piglets slaughtered in the slaughterhouses.

Origin of Findings	Finisher Pigs (*n* = 20,550,072)		Sows (*n* = 484,710)		Piglets (*n* = 94,279)	
	Number	%	Number	%	Number	%
Acute	21,600	0.11 ^b,z^	7426	1.53 ^b,y^	2133	2.26 ^b,x^
Chronic	66,919	0.33 ^a,z^	21,202	4.37 ^a,x^	3877	4.11 ^a,y^
Traumatic	16,712	0.08 ^c,y^	668	0.14 ^c,x^	139	0.15 ^c,x^

^a–c^ percentages in columns lacking a common superscript differ (*p* < 0.001); ^x–z^ percentages in rows lacking a common superscript differ (*p* < 0.001).

**Table 6:** Comparison of the number of individual patho-anatomic findings on the trunk of finisher pigs, sows and piglets slaughtered in the slaughterhouses.

Origin of Findings	Finisher Pigs (*n* = 20,550,072)		Sows (*n* = 484,710)		Piglets (*n* = 94,279)	
	Number	%	Number	%	Number	%
Acute	11,944	0.06 ^b,z^	2098	0.43 ^b,y^	778	0.83 ^a,x^
Chronic	89,576	0.44 ^a,z^	3326	0.69 ^a,y^	799	0.85 ^a,x^
Traumatic	704	0.34 × 10^−2 c,y^	75	1.55 × 10^−2 c,x^	13	1.38 × 10^−2 b,x^

^a–c^ percentages in columns lacking a common superscript differ (*p* < 0.001); ^x–z^ percentages in rows lacking a common superscript differ (*p* < 0.001).

**Table 7:** Comparison of the number of overall changes in finisher pigs, sows and piglets slaughtered in the slaughterhouses.

Findings	Finisher Pigs (*n* = 20,550,072)		Sows (*n* = 484,710)		Piglets (*n* = 94,279)	
	Number	%	Number	%	Number	%
Dwarfism	8401	0.04 ^c,y^	11	0.00 ^c,z^	12,314	13.06 ^a,x^
Emaciation	19,777	0.10 ^b,z^	1626	0.34 ^b,y^	3178	3.37 ^b,x^
Abscesses	35,856	0.17 ^a,z^	12,206	2.52 ^a,x^	2171	2.30 ^c,y^

^a–c^ percentages in columns lacking a common superscript differ (*p* < 0.001); ^x–z^ percentages in rows lacking a common superscript differ (*p* < 0.001).
